# Unraveling pH Regulation of TMEM175, an Endolysosomal Cation Channel With a Role in Parkinson's Disease

**DOI:** 10.1002/jcp.70008

**Published:** 2025-02-04

**Authors:** Tobias Schulze, Oliver Rauh, Gerhard Thiel, Niels Fertig, Andre Bazzone, Christian Grimm

**Affiliations:** ^1^ Walther Straub Institute of Pharmacology and Toxicology, Faculty of Medicine Ludwig‐Maximilians‐University Munich Germany; ^2^ Department of Natural Sciences, Institute for Functional Gene Analytics Bonn‐Rhein‐Sieg University of Applied Sciences Rheinbach Germany; ^3^ Department of Biology Membrane Biophysics Darmstadt Germany; ^4^ Nanion Technologies Munich Germany; ^5^ Immunology, Infection and Pandemic Research IIP Fraunhofer Institute for Translational Medicine and Pharmacology ITMP Munich/Frankfurt Germany

**Keywords:** endosome, lysosome, neurodegenerative disease, PD, TMEM175

## Abstract

Transmembrane protein 175 (TMEM175) is an endolysosomal cation channel, which has attracted much attention recently from academics and the pharmaceutical industry alike since human mutations in TMEM175 were found to be associated with the development of Parkinson's disease (PD). Thus, gain‐of‐function mutations were identified, which reduce and loss‐of‐function mutations, which increase the risk of developing PD. After having been characterized as an endolysosomal potassium channel initially, soon after TMEM175 was claimed to act as a proton channel. In fact, recent evidence suggests that depending on the conditions, TMEM175 can act as either a potassium or proton channel, without acting as an antiporter or exchanger. A recent work has now identified amino acid H57 to be directly involved in gating, increasing proton conductance of the channel while leaving the potassium conductance unaffected. We review here the current knowledge of TMEM175 function, pharmacology, physiology, and pathophysiology. We discuss the potential of this ion channel as a novel drug target for the treatment of neurodegenerative diseases such as PD, and we discuss the discovery of H57 as proton sensor.

## 1 Introduction

Endosomes and lysosomes are critically involved in the regulation of intracellular trafficking, endocytosis, exocytosis, autophagy, cargo logistics, and degradation processes (Xu, Martinoia, and Szabo 2015; Xu and Ren 2015; Grimm et al. 2017; Grimm et al. 2017; Grimm et al. 2018; Rosato, Tang, and Grimm 2021; Prat Castro et al. 2022; Wahl‐Schott and Biel 2023). To maintain their function, in particular, the function of the pH‐sensitive degradative enzymes in the lumen of the lysosomes and the activity of proton‐driven transporters, for example, for the release of amino acids, the luminal pH of lysosomes needs to be tightly controlled and constantly readjusted to about 4.6. In endosomes, the luminal pH needs to be kept at ca. 5.5 (late endosomes) and 6.5 (early endosomes), respectively. A V‐type proton pump is essential to adjust and maintain an acidic pH in both endosomes and lysosomes. Like hypoacidification, hyperacidification can also be a risk for proper endolysosomal function and as such, a proton release mechanism from endosomes and lysosomes appears necessary to prevent hyperacidification events. TMEM175 (Transmembrane protein 175) is a recently discovered endolysosomal cation channel involved in the regulation of lysosomal/endosomal function and, in particular, luminal pH regulation. Thus, TMEM175 was postulated to function as a proton release channel. Disruption in endolysosomal pH can impair trafficking and autophagy, leading to the accumulation of macromolecules such as misfolded proteins, sugars, lipids, and also damaged organelles, a major hallmark of neurodegenerative diseases including Parkinson's disease (PD) and mutations in TMEM175 appear to impact the development of PD (Wie et al. 2021). In the following, we will decipher the knowns and unknowns of TMEM175 function and we will discuss recent developments in our understanding of the mechanism of proton gating of TMEM175.

### 1.1 Function and Pharmacology

Structurally, TMEM175 has two repeats of 6‐transmembrane‐spanning segments, lacking a pore‐forming domain resembling those of the well‐characterized plasma membrane resident voltage‐gated potassium channels. How potassium permeates organelles such as lysosomes and endosomes was largely enigmatic until Cang et al. in 2015 characterized the TMEM175 protein for the first time as an endolysosomal potassium channel, using the endolysosomal patch‐clamp technique. Lysosomes lacking TMEM175 were found to exhibit no potassium conductance, to have a markedly depolarized membrane potential and little sensitivity to changes in [K^+^], and a compromised luminal pH stability, and they showed abnormal fusion with autophagosomes during autophagy (Cang et al. 2015). In 2021, the same group further showed that TMEM175 is activated by growth factors and gated by protein kinase B (AKT; Wie et al. 2021). Most importantly, they also found that a common human variant in TMEM175, M393T is an LOF (loss‐of‐function) variant of the channel associated with an increased risk of developing PD and enhanced accumulation of pathological α‐synuclein, a known hallmark of PD while the opposite was observed for the GOF (gain‐of‐function) variant Q65P, but only under stress conditions such as starvation (Wie et al. 2021). Furthermore, TMEM175 knockout mice showed a loss of dopaminergic neurons and impairment in motor function in mice (Wie et al. 2021). By contrast, in 2022, Hu et al. postulated that TMEM175 mediates a lysosomal proton leak by acting as a proton‐activated, proton‐selective channel on the lysosomal membrane that they called LyPAP. Accordingly, it was proposed that TMEM175's main function is to regulate the flow of proton cations across the endolysosomal membrane, from the endosomal/lysosomal lumen to the cytosol. Thus, TMEM175 would help counterbalance excess H⁺ flux into lysosomes. This balance is critical for the degradation of misfolded proteins such as mutated α‐synuclein, a protein implicated in PD pathology (Hu et al. 2022). Another exciting work which pushed our knowledge about the function of this new endolysosomal ion channel further was the finding by Zhang et al. (2023) that LAMP1 and LAMP2, two lysosomal‐associated membrane proteins, directly interact with and inhibit the activity of the lysosomal cation channel TMEM175. The inhibition by LAMP proteins regulates proton conduction and facilitates lysosomal acidification, while vice versa disruption of the LAMP‐TMEM175 interaction leads to increased lysosomal alkalization, compromising the lysosomal hydrolytic function.

In addition to this protein‐mediated inhibition of TMEM175, 4‐aminopyridine (4‐AP), also a pharmacological inhibitor, has been identified recently, which reportedly binds in the ion conduction pathway of TMEM175 (Brunner et al. 2020; Oh et al. 2022). Of note however, 4‐AP has been described previously as a common blocker also of voltage‐gated potassium channels (Stühmer et al. 1989; Kirsch and Drewe 1993; Grissmer et al. 1994; Yao and Tseng 1994; Schmalz et al. 1998; Coetzee et al. 1999; Gelband et al. 1999; Kerr et al. 2001; Judge et al. 2002; Lang, Mulholland, and Exintaris 2004). Besides 4‐AP, Zn^2+^ was found to block TMEM175 (Cang et al. 2015; Brunner et al. 2020; Zheng et al. 2022). Recently Oh et al. (2024) reported on two new, potentially more selective inhibitors of TMEM175, 2‐phenylpyridin‐4‐ylamine (2‐PPA) and AP‐6. Cryo‐EM structures of human TMEM175 bound by 2‐PPA and AP‐6 revealed that these compounds act as pore blockers. As enhancers of TMEM175 activity, DCPIB, arachidonic acid, and SC‐79, the latter being a small‐molecule AKT activator, were identified (Wie et al. 2021; Bazzone et al. 2023). DCPIB is also a reversible and potent inhibitor of volume‐regulated anion channels (VRAC; Lv et al. 2019); it voltage‐dependently activates the potassium channels TREK1 and TRAAK, and it inhibits TASK1, TASK3, and TRESK (Lv et al. 2019). Clearly, more selective pharmacological tools are needed and are accordingly currently under investigation.

### 1.2 Role of TMEM165 in PD and Therapeutic Potential

In PD, the loss of TMEM175 function or expression compromises lysosomal integrity and function. This dysfunction exacerbates the pathological aggregation of α‐synuclein, contributing to neurotoxicity. Therapeutic strategies targeting TMEM175 may thus offer potential for ameliorating lysosomal dysfunction and slowing PD progression. TMEM175 knockout mice exhibit, besides showing a loss of dopaminergic neurons and an impairment in motor function, also impaired autophagy and an increased susceptibility to cellular stress (Wie et al. 2021). TMEM175 mutations impair lysosomal degradation pathways, leading to the accumulation of α‐synuclein and other toxic proteins, which are key features of PD pathology. Developing drugs that enhance or mimic TMEM175 function could thus help stabilize lysosomal pH and membrane potential, reduce the buildup of neurotoxic proteins, and promote autophagic clearance of cellular waste, thus improving lysosomal function in disease conditions such as PD. Accordingly, small molecules aimed at increasing TMEM175 function might mitigate lysosomal dysfunction in PD patients with TMEM175 mutations but also in cases of neurodegenerative disease phenotypes caused, for example, by other endolysosomal gene defects. Of note, in addition to the abovementioned PD‐associated TMEM175 mutations in humans, Palomba et al. (2023) recently identified further human TMEM175 mutations associated with PD. Palomba et al. (2023) also found that TMEM175 is highly expressed in dopaminergic neurons of the substantia nigra pars compacta and in microglia of the cerebral cortex of the human brain. Using patch‐clamp electrophysiology, Palomba et al. (2023) characterized several of the newly identified point mutations, four of which (TMEM175^R35C^, TMEM175^P308L^, TMEM175^A270T^, and TMEM175^L405V^) showed significantly reduced current densities (LOF). These data suggest that many more mutations in the TMEM175 gene may exist in humans possibly contributing to the pathophysiology of PD patients.

Surprisingly, in a study by Qu et al. (2022) quite some contrary discoveries were made. Qu et al. found that increased TMEM175 function increases the production of reactive oxygen species, disrupts mitochondrial homeostasis, and also inhibits mitophagy. Even more striking, in an MPTP (1‐methyl‐4‐phenyl‐1,2,3,6‐tetrahydropyridine) mouse model of PD, TMEM175 knockout mice showed milder motor impairment and dopaminergic neuron loss compared to the wild type, indicating that loss of TMEM175 exerted a neuroprotective effect, which is inconsistent with the results from previous studies (Jinn et al. 2017; Wie et al. 2021). The authors argued that TMEM175 may play a dual role in different types of PD and that different degrees of TMEM175 deletion may lead to different results, making it possibly relevant to maintain TMEM175 function within a certain range. In sum, these findings seem controversial to previous hypotheses on the role of TMEM175 in PD and strikingly demonstrate that in neurodegenerative disease research not only animal models may bear the risk of not being transferable to human but also that different models within one and the same species may provide seemingly contradictory results. The authors also pointed out that no cases of complete loss of TMEM175 function in humans have been identified so far, with the consequence that it remains unclear whether a complete loss of function causes consistent phenotypes in humans and mice. Of note, however, MPTP treatment induces mitochondria‐initiated cell death leading to the loss of dopaminergic neurons, whereas α‐Synuclein Preformed Fibril (PFF) injections (Jinn et al. 2017) into mouse brains facilitate the aggregation of nondegradable phosphorylated α‐synuclein in neurons. Thus, mechanistically, the two mouse models are very different, with the latter approach (PFF injections) being closer to mimicking common PD pathology in humans, that is, synuclein aggregation (“Lewy bodies”). Another common PD mouse model based on injections of CBE (Conduritol B epoxide) fostering synuclein aggregation might, therefore, be an alternative option to gain more clarity on the role of loss of TMEM175 in PD pathology in the future. Clearly, more research is needed to fully understand the precise pathophysiological consequences of a loss of TMEM175 function as well as the precise mechanisms by which TMEM175 influences endolysosomal activity and function. Furthermore, its interactions with other proteins involved in endolysosomal homeostasis need to be further elucidated including the potential interplay with endolysosomal transporters and other ion channels with pH‐dependent activity.

In sum, while the pathophysiological consequences of TMEM175 dysfunction due to specific point mutations are becoming increasingly evident, for example, more and more TMEM175 mutation‐associated PD cases are being discovered, some uncertainty remains, as outlined above as to whether too much or too little TMEM175 activity or both may have negative implications, in particular for the development or for the progression of neurodegenerative diseases such as PD. Certainly, for effective drug design and drug development, a better understanding of TMEM175's function and mechanism of action is absolutely essential. In the next chapters, we will now discuss specifically recent efforts to further understand mechanistic aspects of TMEM175 action.

### 1.3 Calculated Relative H^+^ and K^+^ Flux Rates in Lysosomes and Endosomes

Since the publication by Hu et al. (2022), there has been debate about whether TMEM175 primarily functions as an H^+^ or a K^+^ channel. Hu et al. (2022) reported a permeability ratio (P_H+_/P_K+_) of 48,000 for TMEM175 using the patch‐clamp technique. This finding was corroborated by Solid Supported Membrane‐based Electrophysiology (SSME), a method that facilitates the assignment of ionic currents to specific ions by applying concentration gradients in the absence of voltage (Bazzone et al. 2023). Consequently, the H^+^ permeability of TMEM175 is approximately 10^6^ times greater than its permeability for K^+^ (Hu et al. 2022, Bazzone et al. 2023). However, to estimate the relative fluxes of these ions, their respective driving forces must also be considered (Bazzone et al. 2023). Five variables influence the driving force and thereby affect the relative K^+^ and H^+^ fluxes through TMEM175: (1) the membrane voltage (Vm) across the lysosomal membrane, which ranges between −20 mV and −40 mV; (2) the cytosolic pH (pHo) maintained at 7.2; (3) the cytosolic K^+^ concentration (Ko) at 140 mM; (4) the intralysosomal K^+^ concentration (Ki), which ranges between 2 mM and 50 mM; and (5) the intralysosomal pH (pHi), set at 4.5. Given that the chemical driving force Δc for K^+^ is approximately 10^4^ times greater than that for H^+^, the H^+^ flux is only 100 times faster than the K^+^ flux when considering the concentration gradients of the two ions alone. When the Vm is also taken into account, K^+^ influx increases further, while H^+^ efflux decreases, resulting in comparable fluxes of H^+^ and K^+^ under physiological conditions. This suggests that both K⁺ and H⁺ conductivities of TMEM175 are functionally relevant at physiological lysosomal pH. To quantify this relationship, we applied the Goldman–Hodgkin–Katz equation (Equation 1) alongside the natural electrochemical driving forces across the lysosomal membrane to derive precise K⁺/H⁺ flux ratios (J_K+_/J_H+_) through TMEM175 (Equation 2) under different physiological conditions.

(1) $$J=P∙\frac{z^{2}F^{2}V_{m}}{\mathrm{RT}}∙\frac{c_{i}-c_{o}∙\text{exp}\left(-\frac{V_{m}\mathrm{zF}}{\mathrm{RT}}\right)}{1-\text{exp}\left(-\frac{V_{m}\mathrm{zF}}{\mathrm{RT}}\right)}$$

Equation 1 (Goldman–Hodgkin–Katz)

(2) $$\frac{J_{K+}}{J_{H+}}=\frac{P_{K+}}{P_{H+}}∙\frac{K_{i}^{+}-K_{o}^{+}∙\text{exp}\left(-\frac{V_{m}F}{\mathrm{RT}}\right)}{10^{-pH_{i}}-10^{-\mathrm{pHo}}∙\text{exp}\left(-\frac{V_{m}F}{\mathrm{RT}}\right)}$$

Equation 2 (Flux ratios)

A negative sign in the J_K+_/J_H+_ flux ratio signifies that the fluxes occur in opposite directions. For our calculations, we used estimated average values of Vm = −30 mV and Ki = 10 mM. In lysosomes with an internal pH of 4.5, approximately three protons are conducted for every K⁺ ion (J_K+_/J_H+_ = −0.3). Notably, fluctuations in Ki and Vm within the physiological range have minimal impact on the flux ratio. However, in late endosomes with pHi = 5.5 and early endosomes with pHi = 6.5, the K^+^ flux dominates with flux ratios of J_K+_/J_H+_ = −2.9 and J_K+_/J_H+_ = −86, respectively. In early endosomes, this implies that 86 potassium ions are transported through TMEM175 for every proton. Due to the relatively small pH gradient in early endosomes, J_K+_/J_H+_ is highly sensitive to variations in membrane potential. For example, when the voltage decreases from −30 mV to −40 mV, the flux ratio changes dramatically from J_K+_/J_H+_ = −86 to J_K+_/J_H+_ = −1635. At a membrane potential of −40.7 mV, the reversal potential for protons is reached, resulting in a theoretically infinite J_K+_/J_H+_ ratio, as only K⁺ ions are conducted. When the membrane potential drops below −40.7 mV, the direction of proton flux reverses, leading to H^+^ influx. Even under conditions designed to minimize potassium flux across the lysosomal membrane (e.g., Vm = 0 mV, Ki = 50 mM), the flux ratio remains in favor of K^+^ (here: J_K+_/J_H+_ = −7.4). These results indicate that potassium flux through TMEM175 is the dominant transport process in early endosomes under all physiologically relevant conditions.

### 1.4 Mechanisms of H^+^ and K^+^ Flux: Interdependence and Gating

The theoretical calculations described above are based exclusively on the permeabilities of H^+^ and K^+^ through TMEM175 and the intrinsic driving forces within lysosomes and endosomes. However, additional factors, such as pH gating and the interdependence of H^+^ and K^+^ fluxes, could modulate the actual fluxes and thereby alter the flux ratios under specific conditions. An important question is whether K^+^ and H^+^ fluxes through TMEM175 occur through the same or separate pathways. If these fluxes share the same pathway, the movement of one ion would inhibit the flux of the other. Utilizing whole‐cell and lysosomal patch‐clamp techniques, multiple studies have now confirmed that, at neutral pH, TMEM175 conductance is dominated by K^+^ ions. However, an acidic endolysosomal pH causes not only a strong increase in TMEM175 conductance but also a shift in ion selectivity toward a preference for H^+^ ions (Hu et al. 2022; Zheng et al. 2022). The observed cross‐dependency of K^+^ and H^+^ fluxes suggests that both ions share a common permeation pathway. Using SSME, the pH dependence of K^+^ flux was further elucidated (Bazzone et al. 2023). Acidification reduces K^+^ flux to about 30% of its full activity, with a pK of 7.0, likely due to pH‐dependent structural changes, as also reported by Zheng et al. (2022). At a pK of 4.5, K^+^ conductivity further drops to zero due to competition with H^+^ flux, which starts dominating in that pH range due to the high P_H+_/P_K+_ ratio.

**Figure 1:**
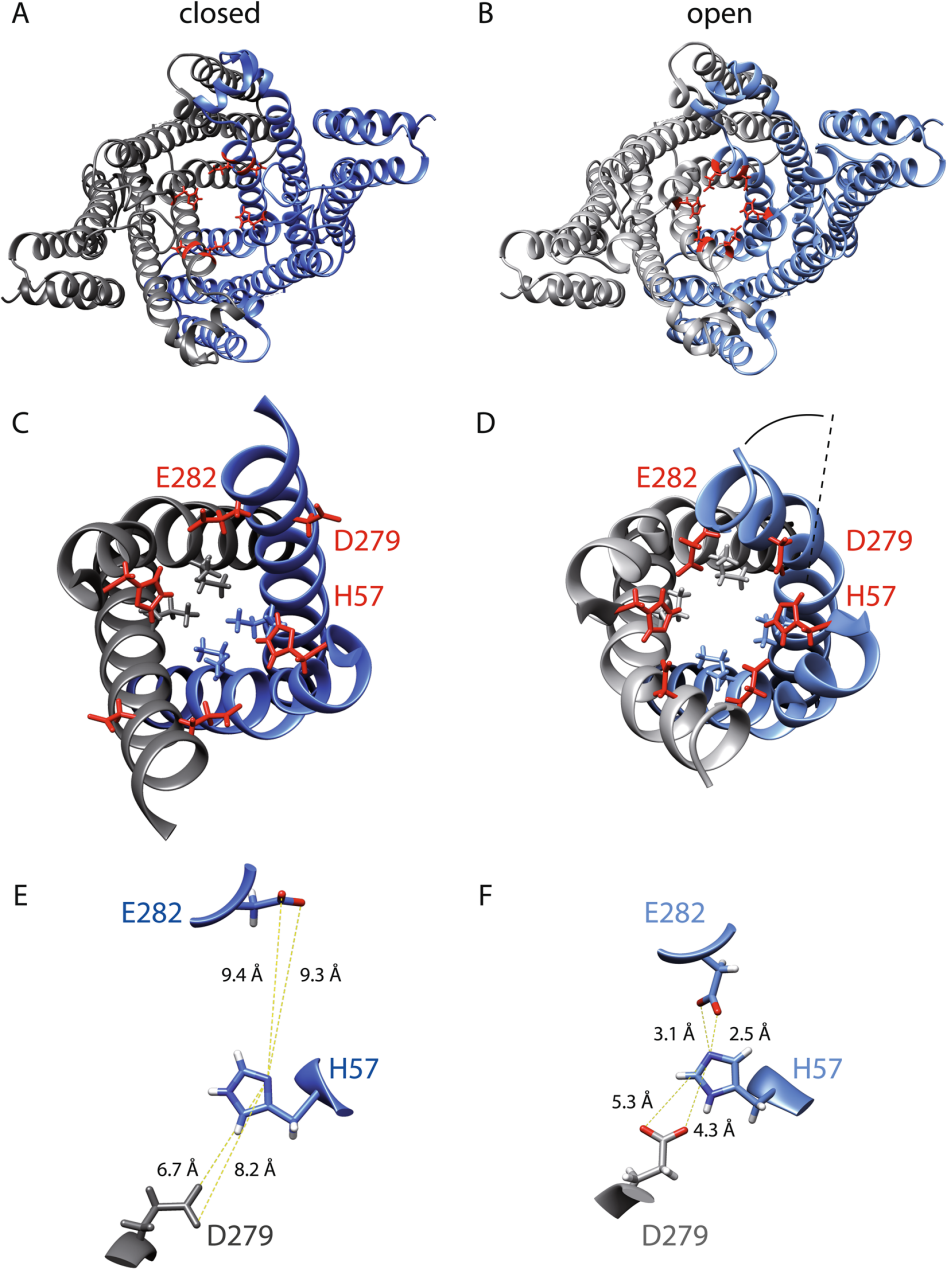
TMEM175 structure showing the stabilization of its open structure by the interaction of H57 with D279 and E282 in a circular manner. (A, B) Cryo‐EM structure of the closed (A) and open state (B) of hTMEM175 in the presence of KCl. One subunit of the TMEM175 dimer is colored gray, and the other one is blue. Darker colors show the closed structure (A), and lighter colors show the open structure (B). H57 and its potential interaction partners (D279 and E282) are highlighted in red. (C, D) Top few on the pore‐lining helices of closed (C) and open structures (D). A kinking of the pore‐lining helices potentially caused by the interaction of the protonated H57 with D279 and E282 leads to the opening of the isoleucine‐gate I46 and I271) and, therefore, of the conduction pathway. (E, F) Distances between the potentially charged atoms of H57 and D279 or H57 and E282 in the closed (E) and open (F) states of TMEM175. Distances are shown as dashed yellow lines labeled with distance values in Å (closed state PDB: 6WCA; open state PDB: 6WC9).

TMEM175 was initially characterized as a leak channel, but recent studies have revealed distinct gating mechanisms. Hu et al. (2022) were the first to demonstrate that TMEM175 functions as a proton‐gated proton channel and established that proton activation occurs at the luminal side of TMEM175. This pH regulation prevents overacidification of the lysosome by activating H^+^ efflux through the channel. Schulze et al. (https://doi.org/10.1101/2024.11.01.621492) could now identify, by employing a mutational analysis based on TMEM175's open and closed conformation structures amino acid H57 to function as luminal pH sensor in TMEM175. In its closed state, the negatively charged residues D279 and E282 are pointing out of the pore. Only the positively charged H57 side chain is facing the pore lumen (Figure 1A,C). Due to the rotation and kink formation of TM7, the negatively charged amino acids are turned toward the pore lumen, where they interact with H57 of TM1 (Figure 1B,D). The interaction of these charged residues seems to be the reason for a helix rotation as well as the kinking of the pore‐lining helices and the resulting opening of the channel gate (Ile46/271; Figure 1D). In addition to this, Oh et al. (2022) showed that Ala48, Met51, and Thr84 are ordering water molecules to stabilize the kinked formation of TM1. Taken together, these interactions may stabilize the open conformation of TMEM175, preferably at acidic pH when H57 is more likely to occur in its protonated state. This hypothesis is in good agreement with the function of TMEM175 in lysosomes (Cang et al. 2015), where the pH is lower than the pK_a_ of histidine. As a consequence, the stimulus that presumably leads to TMEM175 channel opening is the low pH of the lysosomal lumen. The data by Schulze et al. were further corroborated by lysosomal patch‐clamp electrophysiology, providing evidence for the possible dual conductance of TMEM175 in its native context. In support of the results by Schulze et al. Bazzone et al. (2023) found that K^+^ flux is not activated by luminal acidification, indicating that the pH sensor specifically affects H^+^ flux.

The combined data support a critical role of TMEM175 in fine‐tuning endolysosomal pH and provide an explanation for how changes in TMEM175 function may impact the course of neurodegenerative disease development. In fact, as a consequence of these novel findings, direct interaction of small molecules with the pH sensor may be a path forward to developing small‐molecule tools and drugs to interfere with this key amino acid in TMEM175 regulation to modulate its function.

**Figure 2:**
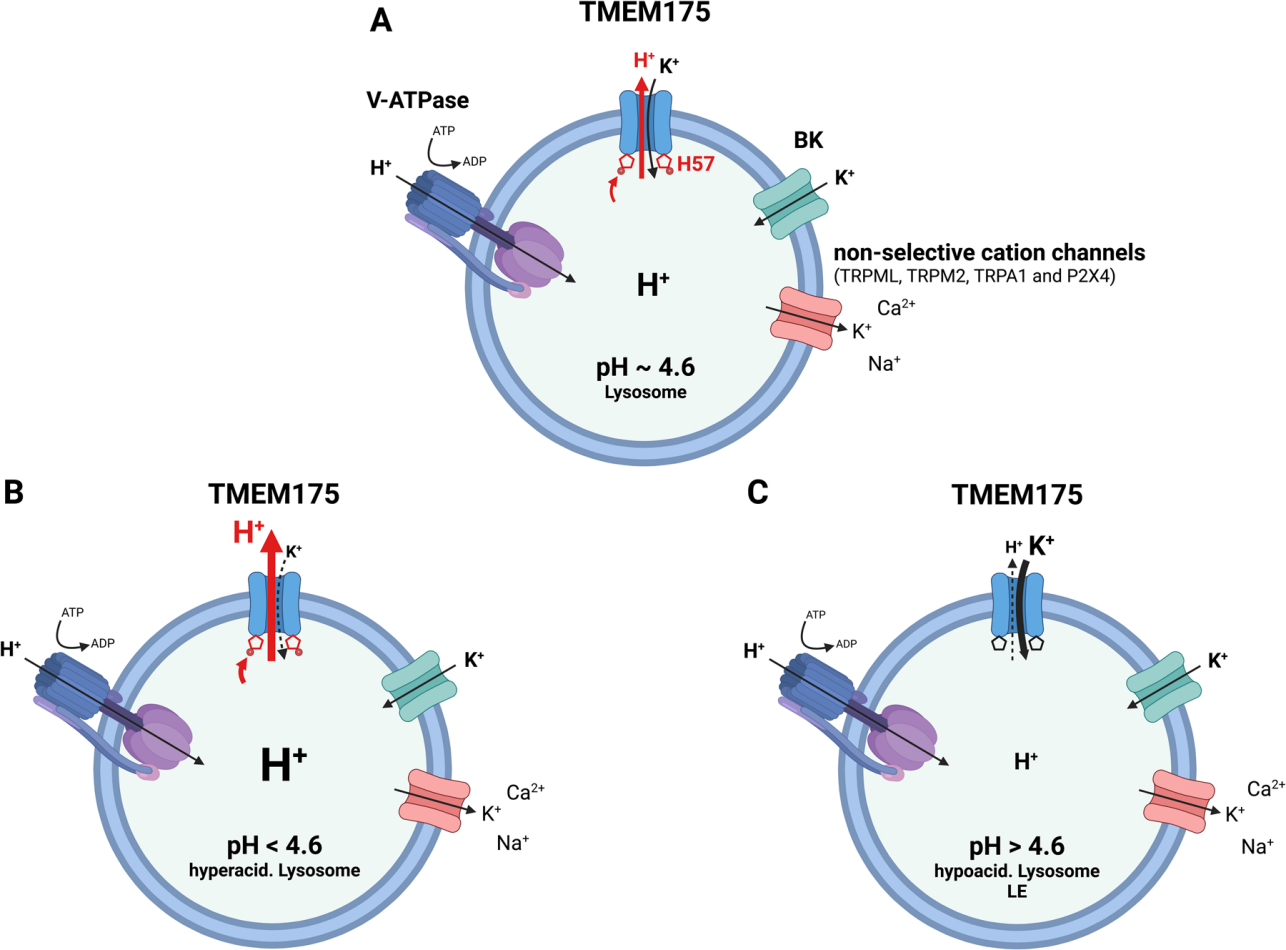
Role of TMEM175 in orchestrating H^+^ and K^+^ homeostasis in endolysosomal vesicles. (A) Function of lysosomal channels at pH 4.6 with H^+^ and K^+^ in equilibrium. TMEM175 conducts a H^+^ current into the cytosol for charge balance of H^+^ accumulation via V‐ATPase in the lysosomes. Here, H57 on the luminal side of TMEM175 acts as a pH sensor. When protonated at acidic pH values, it favors the open conformation of the channel. The K^+^ homeostasis is mainly driven by TMEM175, in addition possibly by BK as well as by nonselective cation channels. (B) In a hyperacidified lysosome, the H^+^ concentration in the lysosomal lumen exceeds the pH optimum of many lysosomal enzymes. In this situation, H57 is increasingly protonated, which in turn increases H+ outward currents and reduces the luminal H^+^ concentration back toward the pH optimum of 4.6. Concomitant with the increase in H^+^ conductance, the K^+^ currents of TMEM175 are reduced at low pH values, and this deficit may be compensated by BK and/or nonselective cation channels. (C) In case of pH values higher than 4.6, as in late endosomes or hypoacidified lysosomes, H57 is increasingly deprotonated, which in turn reduces the H^+^ efflux from the lysosome to the cytosol by TMEM175 while K^+^ influx into the lysosome increases. This favors the V‐ATPase‐driven influx of H^+^ and adjusts the pH back to its optimum (This layout was created using BioRender.com).

In sum, TMEM175 can act as a potassium or proton channel depending on luminal pH and importantly can fulfill either way a protective role regarding endolysosomal pH maintenance, that is, by avoiding hyperacidification on the one hand through the release of excess protons under highly acidic conditions. On the other hand, in its function as major K^+^ conductance across the lysosomal membrane, TMEM175 could likewise support proper maintenance of the luminal pH under less acidic conditions as the influx of K^+^ through TMEM175 into the lumen of the lysosome may activate channels such as, for example, TRPML3 which is known to be more active in high K^+^ to release cations/Na^+^ as counter ions to drive the proton pump to reacidify the lumen of the lysosome (Xu et al. 2023; Figure 2). TMEM175 could functionally interact with, for example, TRPML3 in a similar manner as shown recently for the big‐conductance Ca^2+^‐activated potassium (BK) channel (Xu et al. 2023) with the difference that the activity of TMEM175 would be Ca^2+^ independent. Thus, TMEM175 could protect the luminal pH both from hyperacidification (via increased proton efflux) and hypoacidification (potassium influx dominating, driving regulation of, e.g., cation efflux to enhance luminal re‐acidification). As a consequence, it seems that fine‐balancing TMEM175 activity is key; hence, a complete knockout of TMEM175 might be as detrimental to the maintenance of proper endolysosomal pH as a too strong GOF. This certainly would pose a big challenge for drug discovery as both hypo‐ and hyperactivity of TMEM175 were undesired.

### 1.5 Outlook

TMEM175, TRPML1, TPC2, and other lysosomal ion channels have emerged as promising drug targets in recent years, attracting significant interest from the pharmaceutical industry. Despite their potential, many of these proteins remain understudied, with still limited understanding of their physiological roles and activation mechanisms. It is clear, however, that mutations in these proteins can lead to severe disease phenotypes such as PD or the lysosomal storage disorder Mucolipidosis type IV (MLIV). A key challenge is the parallelization of drug development, aimed at advancing therapeutic strategies, and functional studies to better understand the biological roles of these targets. This is further complicated by the lack of well‐established methodologies for studying lysosomal proteins in their native environment. Regarding TMEM175 specifically, there are multiple open questions. There is a need to elucidate the precise mechanisms governing H^+^ and K^+^ flux regulation, their interplay, their physiological roles, and their respective relevance in maintaining proper cellular function. Additionally, intensifying mutational analyses is essential to clarify the molecular basis of pathologies and disease phenotypes associated with TMEM175 dysfunction. The development of more reliable tool compounds with higher selectivity and efficacy as experimental controls in drug discovery is likewise very critical. Moreover, there is a pressing need for optimized and standardized drug screening methodologies that employ physiologically relevant samples and assay conditions to enhance the translational relevance of findings. Frequently used methodologies to study TMEM175 include lysosomal patch‐clamp and SSME. The advantages and disadvantages of both techniques for studying TMEM175 and lysosomal target proteins in general have been reviewed recently (Bazzone et al. 2023). Patch‐clamp and SSME techniques are complementary. Patch‐clamp allows for precise voltage control, while SSME enables the measurement of substrate‐induced currents, facilitating the distinction between K^+^ and H^+^ currents more easily. Consequently, SSME currents are unequivocally linked to the specific ion species introduced during solution exchange, while patch‐clamp relies on voltage as a less specific driving force, necessitating additional analyses, such as reversal potential measurements, to identify the ion species. However, SSME does not allow for the investigation of voltage‐dependent kinetics. The different stimuli and driving forces between these methods provide distinct experimental contexts, enabling a comprehensive understanding of target protein kinetics, when combined. While significant advancements have been made in the development of automated lysosomal patch‐clamp techniques, SSME challenges the patch‐clamp standard by offering a method that enables electrophysiological recordings of non‐enlarged, native lysosomes under physiological conditions without the requirement of a running cell culture, offering a near‐to‐100% success rate (Bazzone, Barthmes, and Fendler 2017; Bazzone and Barthmes 2020). Several studies employing SSME are currently ongoing, which will lead to advancements in the field of TMEM175 specifically and electrogenic lysosomal membrane proteins in general.

## Author Contributions

Christian Grimm, Andre Bazzone, and Tobias Schulze wrote the manuscript. Oliver Rauh, Gerhard Thiel, and Niels Fertig edited the manuscript. Tobias Schulze designed figures.

## Conflicts of Interest

The authors declare no conflicts of interest.

## Data Availability

The authors have nothing to report.
